# Ordered intermetallic compounds combining precious metals and transition metals for electrocatalysis

**DOI:** 10.3389/fchem.2022.1007931

**Published:** 2022-09-15

**Authors:** Meicheng Yang, Jinxin Wan, Chao Yan

**Affiliations:** ^1^ School of Biotechnology, Jiangsu University of Science and Technology, Zhenjiang, PRChina; ^2^ School of Materials Science and Engineering, Jiangsu University of Science and Technology, Zhenjiang, PRChina

**Keywords:** polymer electrolyte membrane fuel cells, ordered intermetallic alloys, Pt- and Pd-based nanocrystals, electrocatalysts, tunable morphology and structure

## Abstract

Ordered intermetallic alloys with significantly improved activity and stability have attracted extensive attention as advanced electrocatalysts for reactions in polymer electrolyte membrane fuel cells (PEMFCs). Here, recent advances in tuning intermetallic Pt- and Pd-based nanocrystals with tunable morphology and structure in PEMFCs to catalyze the cathodic reduction of oxygen and the anodic oxidation of fuels are highlighted. The fabrication/tuning of ordered noble metal-transition metal-bonded intermetallic PtM and PdM (M = Fe, Co) nanocrystals by using high temperature annealing treatments to promote the activity and stability of electrocatalytic reactions are discussed. Furthermore, the further improvement of the efficiency of this unique ordered intermetallic alloys for electrocatalysis are also proposed and discussed. This report aims to demonstrate the potential of the ordered intermetallic strategy of noble and transition metals to facilitate electrocatalysis and facilitate more research efforts in this field.

## Introduction

Polymer electrolyte membrane fuel cells (PEMFCs) are one of the most promising and feasible energy conversion devices. Due to its small impact on the environment, high power density, and high energy conversion efficiency, it is increasingly favored by researchers. (Gao et al., 2020; He et al., 2020; Prithi et al., 2021; Wan et al., 2021; Kurdin et al., 2022; Liu et al., 2022) However, it is limited by the expensive cost of Pt-based catalysts at the anode and cathode for widespread development. Research found that the ordered intermetallic compounds served as effective electrocatalysts solve the current dilemma. Due to the change of crystal structure and surface structure caused by different atomic arrangement, the intensity and property of adsorption and reaction of chemical substances are changed, thus leading to different catalytic performance. Compared with traditional alloys/single metal nanoparticles, the ordered intermetallic compounds have two advantages: 1) Each crystal position of intermetallic compounds is occupied by a specific atom, so that the structure, geometric effects and electronic effects can be controlled; 2) The formation enthalpy between the noble metal and the second metal M is more negative in ordered intermetallic compounds, and this strong interaction can provide long-term stability and avoid catalyst deactivation. (Xiao et al., 2018a; Wu et al., 2018; Peera et al., 2019; Sun et al., 2019; Mondal et al., 2020; Yuan et al., 2021) The current ordered intermetallic compounds are divided into PtM and PdM, M is a transition metal (M = Cu, Fe, Ni, Co, etc.). Compared with Pd-based ordered intermetallic compounds, Pt-based ordered intermetallic compounds have more in-depth research, and are more accurate for the control of near-surface active sites. Therefore, Pt-based intermetallic compounds generally have higher ORR activities. (Kim et al., 2019; Chen et al., 2020; Liu et al., 2020; Bai et al., 2022; Xing et al., 2022) Thermodynamically, the formation of an alloy or intermetallic compound can be predicted by the change in Gibbs free energy (Δ*G*_mix_) upon mixing. In general, the ordered arrangement of atoms in bimetals should be achieved by thermodynamic control to minimize the Δ*G*_mix_ under given experimental conditions. However, in most cases, bimetallic nanocrystals that should form intermetallic compounds according to thermodynamics end up forming alloy structures with disordered atomic arrangements. The common liquid phase chemical reduction method is difficult to overcome the huge difference in reduction potential between noble metal and 3*d* transition metal precursors, so it is difficult to obtain intermetallic compounds with a single homogeneous structure. In order to promote the transformation of alloys into intermetallic compounds, high temperature thermal treatment is usually required, followed by annealing under the protection of inert gases to maintain the ordered structure among the multi-metals and overcome the kinetic barrier of noble metal atom migration from particle interior to surface. This report mainly summarizes the recent progress of Pt-based intermetallic compounds and gives a brief prospect of Pd-based intermetallic compounds. We also focus on the key structural factors those determine electrocatalytic activity and stability, such as nanoparticle composition, size, morphology, and degree of order. At the same time, related synthetic methods to control the structure of these ordered intermetallic compounds electrocatalysts are proposed.

## Ordered Pt-Based intermetallic compounds

Pt and Pt-based nanomaterials have been used as high-efficiency electrocatalysts for the cathode ORR reaction in fuel cells due to their unique and excellent electronic structure. As early as the 1980’s, related reports indicated that the ordered intermetallic compounds were more active for ORR reactions than disordered intermetallic compounds. However, the improvement in activity and stability of early ordered intermetallic compounds is still limited. And the most critical calcination process for the nanoalloys transition from disordered to ordered structure will cause the sintering of nanoparticles, which will lead to a decrease in activity. (Jin et al., 2017; He et al., 2021; Nie et al., 2021; Kim et al., 2022) With the deepening understanding of ordered intermetallic compounds, intermetallic compounds with new components and structures have been extensively studied. In this part, we will focus on the ordered intermetallic compounds formed between Pt and transition metals, such as PtFe and PtCo, and introduce their remarkable progress.

**FIGURE 1:**
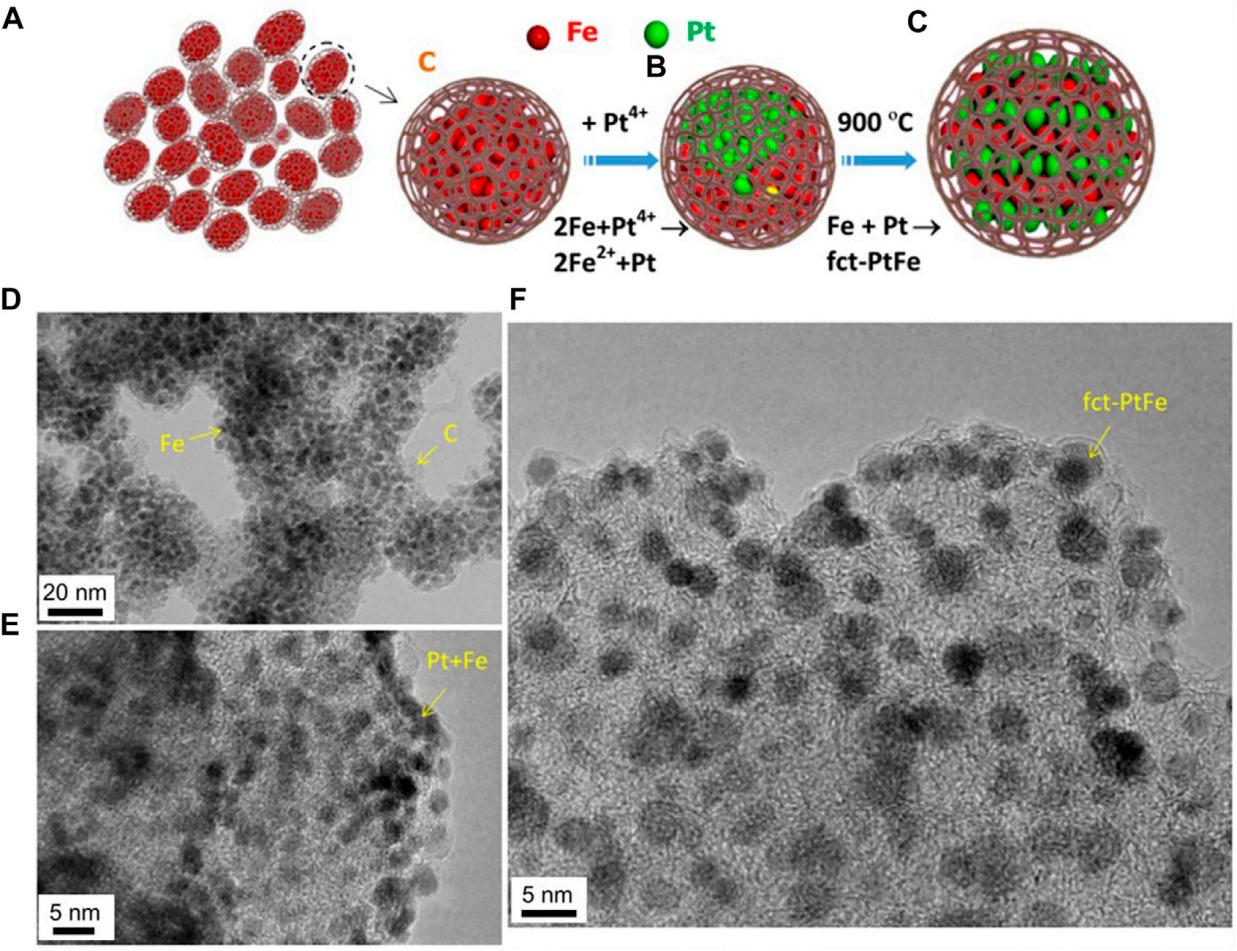
Chemical and structural evolutions. **(A)** Fine Fe particles embedded in carbon matrix. **(B)** Deposition of Pt particles on Fe from the reduction of a Pt precursor. **(C)** Ordered PtFe from structural transformation by heat treatment. **(D)** TEM image of Fe particles embedded in carbon matrix. **(E)** Fine Pt/Fe particles in as‐deposited state. **(F)** Fine PtFe particles entrapped in carbon after heat treatment. (Du et al., 2016) Copyright 2016 Energy and Environmental Science.

In the past five years, a lot of research works have been devoted to the ordered PtFe intermetallic compounds. However, the prepared ordered PtFe intermetallic compounds are all core-shell structures, and the preparation methods of these core-shell structures can be divided into two directions, one is thermal self-reduction, and the other is SiO_2_ template method. It is worth noting that Wang and his colleagues reported a new approach for preparing ordered *fct*‐PtFe nanoparticles, and focused on the control of composition, structure and crystal size. (Du et al., 2016) The specific preparation method is schematically illustrated in Figures 1A–C. The Fe/C powder is prepared by pyrolyzing Fe(CO)_5_ and C_2_H_2_ under certain conditions. Iron carbonyl is easily decomposed to form iron clusters, which catalyze the decomposition of C_2_H_2_ to form carbon atoms. Then carbon atoms are deposited on the surface of the iron clusters, causing the iron clusters to be embedded in the carbon matrix. The final Fe/C powder consists of fine Fe particles (approximately 5 nm or less in size, approximately 55 wt%) embedded in an amorphous carbon matrix (Figure 1D). At the same time, the Pt^4+^ ions from H_2_PtCl_6_∙H_2_O were reduced by Fe nanoparticles and nucleated the deposition of Pt. Then the materials were annealed at 900 °C to obtain ordered *fct*-PtFe nanoparticles. The similar particle size and dispersibility observed on the carbon support indicate that annealing at temperatures as high as 900°C will not cause significant sintering and agglomeration, implying that the pores are beneficial to hold Fe and Pt particles and restrict crystal growth (Figures 1E,F). Compared with Pt-only crystal and other ordered Pt-Fe structures under the same testing conditions, the fully ordered *fct*-PtFe particles show the highest activity and durability in catalyzing ORR in acid. It is due to the geometrical and electronic structures of the ordered PtFe are beneficial for catalytic activity enhancement and chemical stabilization against Fe etching and Pt dissolution. The research on intermetallic compounds is no longer limited to the order of structure. More and more people are beginning to pay attention to the influence of changes in the content of intermetallic compounds on the activity and stability of materials during the ORR reaction. (Zhu et al., 2018a; Zhu et al., 2018b; Li et al., 2022).

**FIGURE 2:**
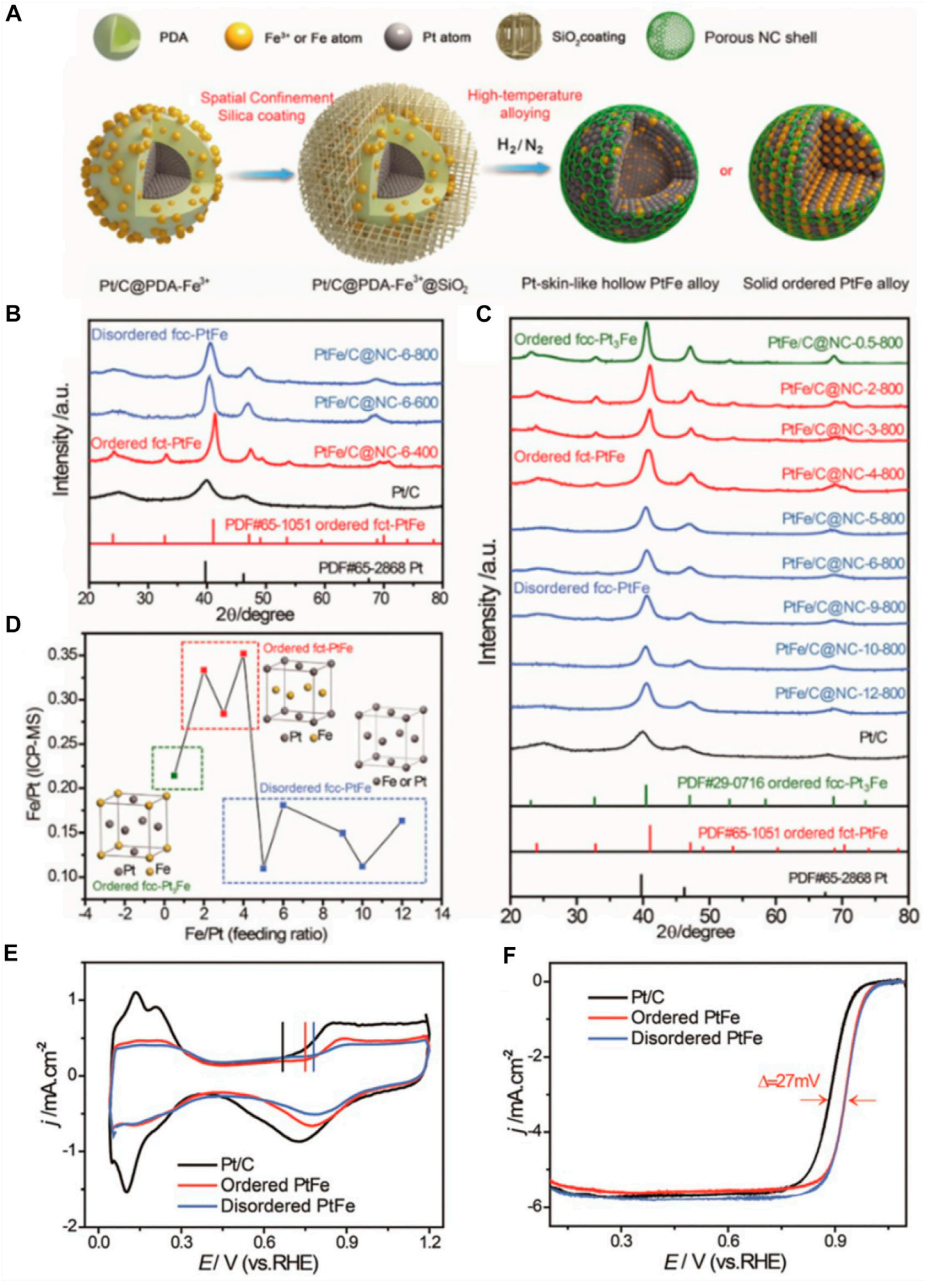
**(A)** Schematic illustration of the thermally driven interfacial diffusion approach. **(B)** Powder X-ray diffraction patterns of PtFe alloy samples obtained with different annealing temperatures at affixed feeding molar ratio of Fe^3+^ to Pt and standard PDFs of Pt (PDF card#65-2,868) and intermetallic PtFe (PDF card #65-1,051), for “PtFe/C@NC-X-T,” where “X” represents the feeding molar ratio of Fe^3+^to Pt and “T” represents the annealing temperature. **(C)** Powder X-ray diffraction patterns of PtFe alloy samples obtained with different feeding molar ratios of Fe^3+^ to Pt at 800 °C and standard PDFs of Pt (PDF card #65-2,868), intermetallic Pt_3_Fe (PDF card #29-0716) and PtFe (PDF card #65-1,051). **(D)** Relationship between the feeding molar ratio of Fe^3+^ to Pt and the corresponding molar ratio of Fe to Pt, as determined by ICP-MS. **(E)** CV curves of typical ordered PtFe (PtFe/C@NC-3-800), disordered PtFe (PtFe/C@NC-9-800) and Pt/C catalysts recorded in N_2_-purged 0.1 M HClO_4_ solution at a scan rate of 50 mV s^−1^. **(F)** ORR polarization curves of typical ordered PtFe (PtFe/C@NC-3-800), disordered PtFe (PtFe/C@NC-9-800) and Pt/C catalysts recorded in O_2_-saturated 0.1 M HClO_4_ solution at room temperature with a sweep rate of 10 mV s^−1^ and a rotation rate of 1,600 rotations per min. (Zou et al., 2019) Copyright 2019 Nanoscale.

Ordered PtFe alloys all have Fe precipitation in practical applications. In order to prevent such phenomena from occurring, core-shell structures are established. At the same time, this core-shell structure generally has a thin layer of Pt. Wei group have developed a thermally driven interface diffusion alloying route that allows solid Pt nanoparticles (NPs) directly transformed *in situ* on carbon (Pt/C) into Pt-skin-like hollow PtFe alloy or structured intermetallic PtFe alloy (Figure 2A). (Zou et al., 2019) As a result, the PtFe alloy NPs are encapsulated *in situ* with the thin porous nitrogen-doped carbon (NC) shell. Through the research results of powder X-ray diffraction (XRD) and inductively coupled plasma mass spectrometry (ICP-MS), Wei and his colleague explored the crystal structure of PtFe alloys annealed at different Fe^3+^ contents and temperatures. The XRD patterns of the PtFe alloys obtained at different annealing temperatures and affixed feeding molar ratio of Fe^3+^ to Pt can be seen in Figures 2B,C. The transformation from disordered to ordered structure is often carried out at a specific temperature. As the annealing temperature continues to increase, the formed ordered intermetallic compound *fct*-PtFe phase disappears, resulting in disordered face-centered cubic (*fcc*) PtFe Alloy phase (PtFe/C@NC-6-600 and PtFe/C@NC-6-800). It is worth noting that the low raw material ratio of Fe^3+^ and Pt is more conducive to the formation of ordered intermetallic compounds. The competition between the chemical order energy and the surface segregation energy will lead to the phase transition observed at different annealing temperatures and Fe^3+^ contents. In addition, high annealing temperature or high Fe^3+^ content will destroy the equilibrium arrangement of Pt and Fe atoms on the formed ordered PtFe lattice sites, thereby causing surface segregation. Therefore, it can be concluded that the structural evolution of the Pt to PtFe alloys can be precisely controlled by adjusting the Fe^3+^ content and the annealing temperature, which is guided by the competition between the chemical order energy and the segregation energy. As determined by ICP-MS, the disordered PtFe alloys obtained with a higher feeding molar ratio of Fe^3+^ to Pt had a low Fe content in the final products because of surface segregation, while the ordered PtFe alloys obtained with a lower molar ratio had a high Fe content in the final products (Figure 2D).

What’s more, as shown by the vertical line in Figure 2E, compared with the Pt/C catalyst, the corrected onset potential for Pt oxidation in the ordered PtFe and disordered PtFe catalysts further illustrates that the ordered intermetallic compounds reduce the absorption of intermediate oxygen-containing species. From the typical ORR polarization curves (Figure 2F), it can be clearly seen that the initial potential and half-wave potential of ordered PtFe/C intermetallic compounds are better than that of disordered intermetallic compounds. Meanwhile, Wei and co-workers reported a preparation method to effectively prevent the sintering and migration of ordered PtFe nanoparticles during high-temperature annealing using a well-structured polydopamine inner shell and silica outer closed space, by which can directly synthesized ordered small-scale PtFe alloys embedded in thin porous N-doped carbon protective shells. (Zou et al., 2019) The ordered PtFe alloy catalysts exhibited admirable ORR performance, which is attributed not only to the special geometric and electronic structure of the ordered PtFe NPs, but also to the thin NC-coated shell. The protection of NC shell and the formation of ordered structure provided enhanced stability. This work not only demonstrated the benefit of doping to enhance stability, but also further enhanced the catalytic activity by incorporating an ordered alloy strategy, providing an effective strategy for fuel cell development.

The improved performance of ordered PtFe intermetallic compounds can be attributed to three reasons. First, the incorporation of Fe causes the Pt lattice to gradually shrink, thereby shortening the distance of the Pt-Pt bond, which is conducive to improving the catalytic activity. (Wang et al., 2019a; He et al., 2019) Second, the intermetallic structure in *fct*-PtFe provides some ideal catalytic surfaces around each crystal. Pt is located on the top of the crystal and Fe is located below the Pt layer, which is essential for catalytic strengthening. (Chung et al., 2015; Liu et al., 2015) Third, the highly *fct*-ordered structure and strong *d*-interaction between Fe and Pt tends to be stable, especially the reaction of Fe and Pt in acidic solutions. (Hu et al., 2020; Yang et al., 2022) However, it is worth noting that metal leaching occurs under high-potential electrochemical conditions. Previous simulation studies have shown that in the *fct*-PtFe structure, Fe and Pt interact strongly through spin-orbit coupling, and the hybridization of Fe 3 days and Pt 5 days states makes the chemical structure of *fct*-PtFe more stable. (Jung et al., 2017)

Since annealing reduction is a reliable and facile method to transform disordered phase to ordered phase, suitable calcination temperature is a key factor for the synthesis of ordered intermetallic compounds. In the common annealing methods, low temperature is often not conducive to the formation of ordered structures, but will cause sintering. (Jung et al., 2020; Cao et al., 2021) At the same time, a suitable calcination temperature may also lead to the formation of core-shell structures. In addition, such core-shell structures are generally oxides of transition metals. Gan’s group reported an ordered PtCo catalyst with a core-shell structure synthesized at a suitable temperature. (Wang et al., 2019b) The thickness of the shell is approximately 3–5 nm. The ordered PtCo intermetallic compounds differ from ordered PtFe intermetallic compounds in that the Co distribution in the ordered PtCo intermetallic compounds is more uniform with fewer surface oxides and carbon layers, which is reasonable because that Co does not have the strongest oxygen affinity as Fe does. Therefore, the ordered PtCo intermetallic compounds are not easy to form an oxide layer as ordered PtFe intermetallic compounds, which will inevitably lead to the subsequent better oxygen reduction performance than that of ordered PtFe intermetallic compounds.

Branko innovatively discovered and developed an ordered face-centered tetragonal (*fct*) Pt-based intermetallic compound with high activity and stability. (Jung et al., 2020) The experiments started with the successful introduction of Co and N into graphitized carbon (GC) supports by pyrolysis of the Co and N precursor mixture. Co protected with N-doped graphitic carbon shell was later used to form PtCo catalyst. Therefore, after deposition of Pt by a modified polyol method (Pt/Co@CN/GC), a controlled heat treatment was performed under a reducing atmosphere to form PtCo/NGC. Due to the formation of a chemically stable *fct* structure, which alleviates the Co dissolution and controls the surface oxide thickness, as well as the high carbon oxidation resistance of Co@CN/GC, the PtCo/NGC catalyst exhibited improved electrocatalytic activity and stability towards ORR as a cathode catalyst compared with the commercial Pt/C and commercial PtCo/GC catalysts.

## Ordered Pd-Based intermetallic compounds

So far, Pt-based nanomaterials are still the most commonly used catalysts for PEMFC. However, the wide application of Pt is limited due to its rarity and high cost. Researches show that Pd-based electrocatalysts are viable alternatives to Pt-based electrocatalysts. Pd-based electrocatalysts have a certain high catalytic activity and stability, and the cost is much lower than that of Pt. Meanwhile, some strategies to promote the catalytic performance of Pt can also be applied to the Pd system. Previous studies on Pd-based catalysts mainly focused on disordered structures. The following describes the development of ordered Pd-based catalysts in recent years. As mentioned earlier, ordered structures have unique advantages in achieving higher electrocatalytic activity and stability than disordered structures. (Xiao et al., 2018b; Wu et al., 2018; Kang et al., 2020; Luo et al., 2020; Gong et al., 2021)

**FIGURE 3:**
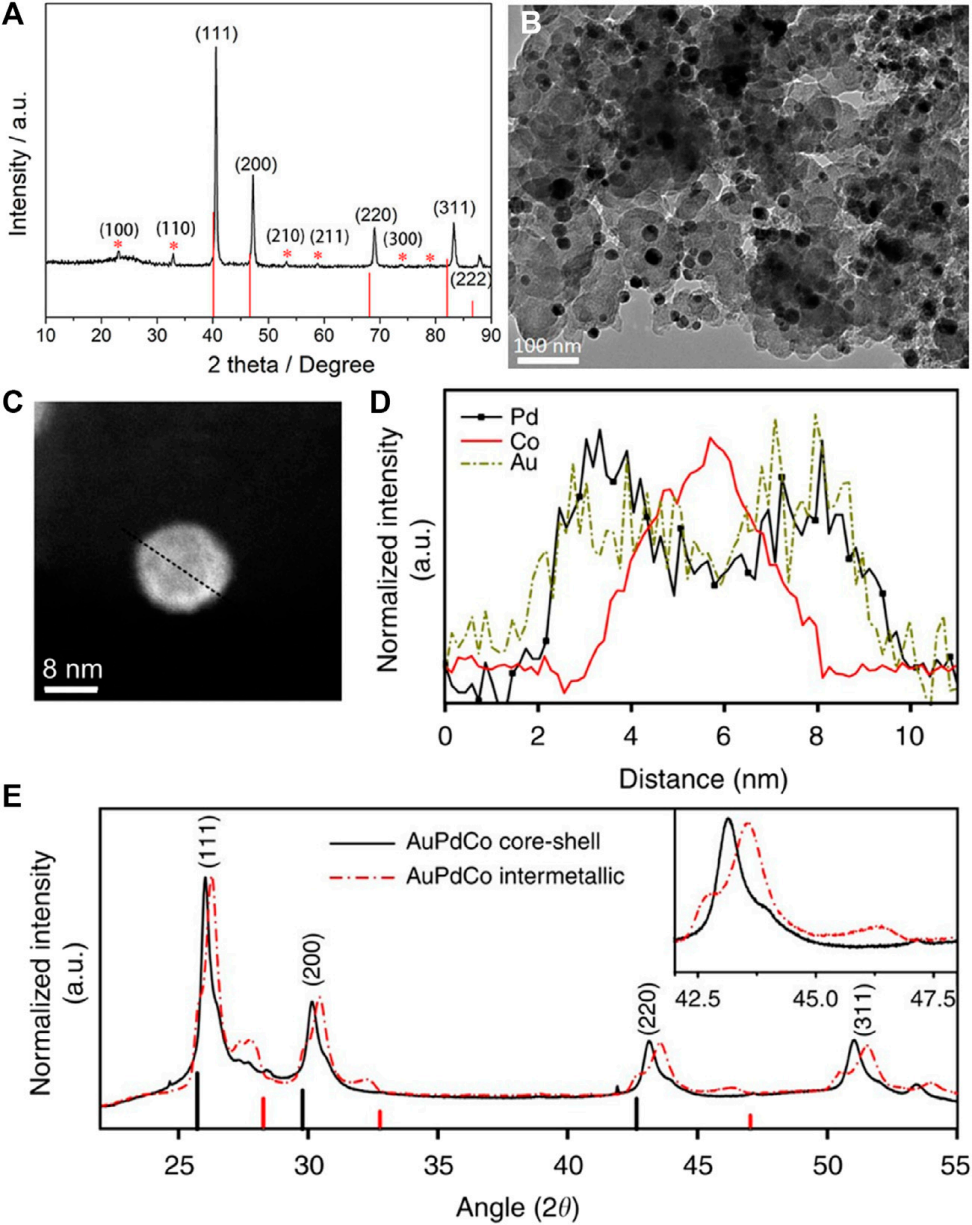
**(A)** XRD pattern of the ordered Pd_3_Fe/C; drop lines correspond to Pd (JCPDS No. 46-1,043). **(B)** TEM image of the ordered Pd_3_Fe/C. (Liu et al., 2018) Copyright 2018 Nano Research. **(C,D)** High angle annular dark-field-STEM image of a core-shell AuPdCo nanoparticle with its corresponding EELS line-scan profiles. Scale bar, 5 nm. **(E)** XRD patterns for core-shell and intermetallic AuPdCo nanoparticles. The black and red vertical lines indicate the (111), (200), and (220) peak positions of pure Pd and Co reflections, respectively. The inset shows the enlarged region of the AuPdCo (220) diffraction peaks. (Kuttiyiel et al., 2014) Copyright 2014 Nature Communications.

Inspired by the synthesis of ordered PtFe intermetallic compounds, more and more researchers strive to prepare cheaper ordered PdFe as an alternative to Pt-based electrocatalysts. Just like ordered PtFe, the successful synthesis of ordered PdFe is also demonstrated by the existence of “ordered peaks” in the XRD patterns. (Liu et al., 2018) It can be clearly seen in Figure 3A that the five typical diffraction peaks assigned to the (111), (200), (220), (311), and (222) planes have a positive shift relative to the standard plane of *fcc*-Pd phase (JCPDS No. 46-1,043), indicating the formation of PdFe alloys. In addition to these five typical diffraction peaks, another six superlattice peaks corresponding to the (100), (110), (210), (211), (300) and (310) planes (so-called “ordered peak”) were detected, which are consistent with the ordered intermetallic compound Pd_3_Fe (JCPDS No. 65-7,280). Most of the particle size of the ordered Pd_3_Fe intermetallic compounds can reach ∼11 nm (Figure 3B). At the same time, the atomic ratio of the ordered PdFe intermetallic compounds prepared by current researchers is all 3:1, which is inconsistent with ordered Pt-based nanomaterials. In ordered PdFe intermetallic compounds, it is difficult to form an oxide layer due to the protection of Pd, so it is difficult to form a core-shell structure. It is worth noting that there are reports that ordered PdFe intermetallic compounds have excellent catalytic activities in both basic solution (0.1 M KOH), acidic solution (0.1 M HClO_4_) and formic acid solution. (Xiao et al., 2018b; Ji et al., 2019)

Co is another widely used transition metal to promote catalytic properties of Pt and Pd. However, reports on the electrocatalysis of ordered PdCo intermetallic compounds are very limited, which may be due to the difficulty in obtaining nanoscale ordered intermetallic PtCo phases by general annealing processes. (Kuttiyiel et al., 2014; Zeng et al., 2018; Li et al., 2020; Hu et al., 2022) This difficulty is addressed by a simple approach of core-shell structured PdCo nanoparticles, which can be further transformed into ordered intermetallic phases by addition of Au atoms and subsequent annealing. At high temperature, PdCo nanoparticles undergo an atomic structural transition from core-shell to rare intermetallic ordered structure, which forms stable (111), (110), and (100) planes by adding Au atoms. (Kuttiyiel et al., 2014) As shown in Figure 3C, the synthesized nanoparticles possess a core-shell structure. Meanwhile, the electron energy loss spectroscopy (EELS) line scan profile of a single PdCoAu NP confirms a Co-rich core covered by a PdAu-rich shell (Figure 3D). The XRD patterns of the two powder samples (Figure 3E) have four peaks corresponding to (111), (200), (220), and (311), which closely match the peaks expected for face-centered cubic structure. The intermetallic phase of the nanoparticles may have contributed to further shrinkage, with peaks shifted to much higher angles than core-shell nanoparticles. The small shoulder at the 2*θ* angle of 42.6^o^ corresponding to the (220) reflection (inset of Figure 3E) is due to the synergistic effect of the surface AuPd. The addition of Au atoms can promote structural ordering of PdCo nanoparticles under elevated temperature. AuPdCo-intermetallic nanocatalyst shows comparable ORR activity with commercial Pt/C but much better long-term stability in alkaline medium. The increased activity and durability of the catalyst is because of the multiple facets and the ordered structure. The ORR activity is also affected by the pH and leads to different mechanism in acid and alkaline media. In acidic media, nanoparticle size effect has an important role in increasing the electrochemical surface area, thus increasing the catalytic activity, but in alkaline media, only little changes were due to nanoparticle size effects.

## Conclusion and outlook

PEMFC is an ideal alternative to traditional energy conversion technology but cannot be afforded due to its high cost. Ordered Pt-based and Pd-based intermetallic compounds with controllable size, composition, and morphology have been widely studied as highly active and stable electrocatalysts for PEMFC. The ordered intermetallic compounds exhibit significantly enhanced activity and stability due to their strong electronic interactions and uniform active sites. The well-defined composition and elemental distribution of ordered intermetallic structures make them more suitable for studying the intrinsic structure-property relationships. However, it is still a great challenge to overcome the thermodynamic barrier caused by the difference of electrode potential between metal precursors, solve the problem of dynamic growth caused by the difference in particle size among polymetals, and synthesize Pt-based and Pd-based intermetallic compounds with ordered structures by simple and effective methods to improve the electrocatalytic activity and stability of catalysts. Considering the remarkable progress discussed in this article, we confidently expect more intensive work to be carried out in this area.
